# The Book World of Medicine and Science

**Published:** 1900-08-11

**Authors:** 


### The Book World of Medicine and Science.

Tuberculosis: Its Nature, Prevention, and Treatment. By A. Hillier, B.A., M.D., C.M. Pp. 242, 8vo. 31 Illustrations. (London: Cassell and Co., Limited. 1900. Price 7s. 6d.)

Of the many books whose inception is due to the new crusade this is perhaps the best. Dr. Hillier, who in the term of his South African residence has had great experience, has very wisely refused to limit his scope to that of pulmonary tuberculosis, and has instead reviewed the whole subject of tuberculosis in its manysidedness. The result is that we have a book capable no doubt of some expansion, but adequate and judicial.

Dr. Hillier devotes his first chapter to a general sketch of tuberculosis; in succeeding chapters he deals with the different clinical forms, and with the serious problems of transmission from man to man, of transmission from animals to man, of prevention in everyday life, and of prevention by legislation and public action. The treatment of tuberculosis is outlined in a comprehensive and sagacious essay, and the book is completed by some account of the national movements against tuberculosis. This outline will serve to give some idea of the task Dr. Hillier has set himself, and we have little but praise for the manner in which he has accomplished it. When we consider the small compass of this little work we are surprised how few facts of importance have been omitted and how much Dr. Hillier has incorporated into these pages. Many excellent illustrations are given of sanatoria and sun-traps, and Dr. Hillier has also seen the possibilities of "Rowton Houses," and has employed photographs of them to show what can be done for the masses of our great towns. One fact that Dr. Hillier mentions deserves notice, that we may realise how in some matters sociological we are dropping behind the Germans. Certain insurance societies with which compulsory insurance is, for certain classes in Germany, enforced by law have actually found it to their advantage to erect sanatoria and give their clients the benefit of gratuitous treatment therein. Dr. Hillier has provided an index to this work, and an appendix containing some valuable information. We know of no work which will more effectually unite medical men in the fight against tuberculosis, and furnish them with logical weapons against reactionaries than this of Dr. Hillier's.

Anæsthetics: Their Uses and Administration. By D. W. Buxton, M.D., B.S. Pp. 318. 8vo. Illustrated. Third edition. (London: H. K. Lewis. 1900. Price 6s.)

Dr. Dudley Buxton's well-known work having been for some time out of print, the issue of a third edition has been determined on and the opportunity seized to give some account of, amongst other innovations, the practices of giving nitrous oxide and ether with oxygen, and the infiltration method of local anæsthesia. For the sake of those unacquainted with Dr. Buxton's manual we may say that, without exhausting his subject, he traverses most of the ground that should be familiar to the student. Systematic completeness would overburden such a book, and it is not to be wondered at, but rather to be commended, that Dr. Buxton should have chosen for description those methods and instruments that his great experience has led him to prefer. It would be, we think, stating Dr. Buxton's position fairly to say that, in the first place, and in the absence of contra-indicating conditions, he prefers to employ ether, and from the apparatus known as the "large clover." In the matter of chloroform administration he inclines to the giving of measured quantities, as from Junker's bottle; and, while persuaded of the practical importance of watching the respiration, dissents from some of the more theoretical views of the Hyderabad Commission. Perhaps logically he is a little severe in asking for proof of the non-occurrence of primary cardiac failure, inasmuch as the Commission can only state that they have met with no such event. Dr. Buxton enters upon the purely scientific side of his subject with considerable detail, which must be of profit to the student, and is not niggard in the imparting of practical "tips." There are a few rough places in the generally even track of his pen, and a few slight omissions: it might here be stated that, not glycosuria, but the presence of glycuronic acid in the urine, may follow the giving of chloroform. And we do not think that Dr. Buxton has omitted purposely notice of Dr. Ringer's treatment and prophylaxis of ether bronchitis—the giving of belladonna. Of especial value are Dr. Buxton's remarks on the ethics, medical and legal, of anæsthetisation. Indeed, on every point he speaks with the authority that his great experience gives him, and demands the attention that his scientific position naturally compels.

Handbook of Physiology (Kirkes' Handbook). By W. D. Halliburton, M.D., F.R.S. (Sixteenth edition. London: John Murray. 1900. Price 14s.)

Our old yet ever young friend, Kirkes' "Handbook of Physiology," appears again, and this time in a sixteenth edition. Under such circumstances we hardly need say much beyond congratulating Professor Halliburton on his success in maintaining the popularity of the work, while at the same time keeping it accurately in touch with the advances of the ever-advancing science with which it deals. The popularity which Kirkes' Handbook always had among students even in the days when, in its old form, it was hardly regarded among teachers as an authoritative exposition, was no doubt due, on the one hand, to its being a teaching book rather than a mere work of reference, and on the other to the fact that it contained enough of anatomical and historical detail to render the tale it told a fairly complete one, and one which could be followed without reference to other volumes; and notwithstanding the new features, the accuracy of statement, and the scientific up-to-dateness which Professor Halliburton has introduced into the more recent editions, the book still retains its old characteristics and apparently its old popularity. To the medical student this self-contained completeness is, perhaps, not so essential, seeing that in other books he has at hand the necessary information, although even to him it is no small advantage to have everything together under one cover. But to those who, although not aiming at medicine, yet wish to obtain a good working knowledge of human physiology, this book stands *facile princeps* as a guide to knowledge, leading one as it does from the simplest problems to the most abstruse, and always explaining things as it goes on. The illustrations are very numerous, and are admirable throughout, and the colour printing, which is largely employed even on the printed page, adds greatly to the teaching efficiency of the drawings. In every department the book seems to have been brought well up to present-day knowledge, and it is to be noted that in the portion devoted to the nervous system the more modern views are thoroughly accepted and explained. In every way the book is to be relied on, and, although it is manifestly impossible to compress all that is known on the subject into a volume of between 800 and 900 pages, there can be no doubt that it fulfils its purpose as a student's handbook with great perfection.

